# Finnish Registry-Based Protocol for Screening and Management of Fatigue and Cognitive Problems in Multiple Sclerosis: Observational Study

**DOI:** 10.2196/67990

**Published:** 2025-08-22

**Authors:** Päivi Hämäläinen, Elina Lämsä, Matias Viitala, Hanna Kuusisto, Marja Niiranen, Sari Avikainen, Juha Puustinen, Mervi Ryytty, Juhani Ruutiainen, Merja Soilu-Hänninen

**Affiliations:** 1 StellarQ Ltd, Eerikinkatu 3 A, Turku, 20100, Finland, 358 408671566; 2 Finnish Neuro Society, Masku, Finland; 3 Department of Health and Social Management, University of Eastern Finland, Kuopio, Finland; 4 Novartis Finland, Espoo, Finland; 5 Department of Neurology, Tampere University Hospital, Tampere, Finland; 6 Neuro Center, Neurology, Kuopio University Hospital, Kuopio, Finland; 7 Neurology, Nova Hospital in Central Finland, Jyväskylä, Finland; 8 Unit of Neurology, Satasairaala Central Hospital, Satakunta Wellbeing Services County, Pori, Finland; 9 Medical Research Center and Neurocenter, Oulu University Hospital, Oulu, Finland; 10 Research Unit of Clinical Medicine, University of Oulu, Oulu, Finland; 11 Clinical Neurosciences, University of Turku, Turku, Finland; 12 Neurocenter and Division of Clinical Neurosciences, Turku University Hospital, Turku, Finland

**Keywords:** multiple sclerosis, electronic health records, digital health, patient-generated data, cognition, fatigue

## Abstract

**Background:**

Digital patient registries are actively used to monitor long-term diseases. However, their potential in symptom management remains underused.

**Objective:**

This study aimed to report on the Finnish registry-based protocol to screen and manage cognitive symptoms and fatigue in multiple sclerosis (MS). Data on a sample collected during the first 2 years are presented.

**Methods:**

At the beginning of 2021, a Finnish protocol to screen and manage patient-perceived concerns related to cognition and fatigue, together with self-assessment of disease severity, symptoms, and quality of life (QoL) annually, was introduced. The Symbol Digit Modalities Test (SDMT), the Multiple Sclerosis Neuropsychological Questionnaire (MSNQ), the Fatigue Scale for Motor and Cognitive Functions (FSMC), as well as the Patient-Reported Expanded Disability Status Scale, the Visual Analog Scales, and the Euro QoL-5 Dimension were implemented into the Finnish MS registry. To support symptom management, patients were offered feedback reports based on the results of the FSMC and the MSNQ. The implementation of the protocol was evaluated in 5 Finnish wellbeing services counties.

**Results:**

Our sample from the beginning of 2021 to the end of 2022 includes data on 430 patients. A total of 86 (20%) patients have been assessed with the SDMT, whereas 329 (76.5%) patients have filled out the FSMC, and 172 (40.0%) patients have completed the MSNQ. The mean SDMT score is 49.0 (SD 13.56), MSNQ score is 35.3 (SD 9.39), total FSMC score is 63.0 (SD 22.49), and subscores for motor and cognitive fatigue are 31.6 (SD 11.43) and 31.5 (SD 11.68), respectively. The SDMT did not correlate with the MSNQ or the FSMC. Instead, the SDMT, MSNQ, and the FSMC correlated significantly with QoL.

**Conclusions:**

Fatigue and cognitive problems have an effect on QoL. In our preliminary sample, patient reports of cognitive problems and especially fatigue were conducted more frequently than the objective evaluation of processing speed. Although the Finnish MS registry offers a digital platform for the systematic screening of fatigue and cognitive problems, further education is needed to support the implementation of the protocol.

## Introduction 

Multiple sclerosis (MS) registries are in active use in many European countries [1], and the need to develop registry-based data collection is recognized worldwide [2]. Registries help with patients’ follow-up and care [3,4]. There is a need to increase patient involvement and the collection of patient-generated data. Tools to identify and manage the wide range of patient-perceived MS-related concerns are needed. Moreover, there is a special need for early recognition of symptoms and factors that affect productivity and quality of life (QoL).

According to a mapping survey, there are 19 MS registries in Europe [1]. Patient-reported outcomes (PROs) are included only in 7 registries. One of those is the Finnish MS registry with a unique patient interface. The Finnish MS registry was launched in 2014 to enable digital treatment monitoring, systematic follow-up of incidence and prevalence, as well as the course of the disease [5]. At present, the registry is used in 18 out of 21 of Finland’s wellbeing services counties, including all 5 university hospitals. A patient interface for PROs was created in 2017, allowing patients to contribute health data on their symptoms, QoL, and overall well-being to inform health care professionals (HCPs) and to support patient-centered treatment decisions [6,7]. The PROs implemented in the patient interface include validated patient-reported outcome measures (PROMs) as well as more informal PRO data.

Fatigue has been presented as the most common symptom of MS, with a rate of up to 80% [8-10], whereas approximately 50% of patients with MS have been shown to have cognitive deficits [11]. The negative effects of cognitive problems and fatigue on working ability have been shown in registry-based studies [12,13]. Self-perceived cognitive problems and fatigue have shown a linear relationship with days on sick leave and a decrease in weekly working hours and self-perceived working ability [12]. Furthermore, impaired processing speed as evaluated by the Symbol Digit Modalities Test (SDMT) has been found to be related to decreased work productivity as measured by days on sick leave and total annual income [13]. Cognitive deficits and fatigue, as invisible symptoms, often remain underdiagnosed, and treatment options are considered too late to promote working ability. Despite these findings, patient reports on fatigue or cognitive problems are not systematically included in MS registries [1]. Because the underlying mechanisms of fatigue are multifaceted and no clear definition for the symptom exists, the assessment is particularly challenging and relies heavily on patient report [8]. The fact that fatigue and cognitive concerns have been reported to be confounded with depression and problems with mood further complicates the assessment of the symptoms [14]. At the same time, the detrimental effects of fatigue and cognitive concerns on employment call for early diagnostics and treatment.

In 2020, a Finnish initiative called Invisible Symptoms in MS was presented to meet the need to consider cognitive symptoms and fatigue systematically and in a cost-effective way already at the early phases of the disease. The primary aims of the initiative were to promote early and holistic evaluation and treatment of MS and to offer patients tools to evaluate and manage fatigue and cognitive problems by themselves. The objective of this paper is to describe the protocol to screen and manage fatigue and cognitive problems by using the Finnish MS registry. Furthermore, descriptive statistics are reported on the data collected during the first 2 years following the implementation of the procedure, based on a sample from 5 well-being services counties.

## Methods

### Finnish MS Registry

The Finnish MS registry is a browser-based registry for public health care organizations [5]. The software service is provided by StellarQ Ltd. The use of the registry is voluntary, and each well-being services county has decided individually whether to acquire it. At the time of the study, 17 out of 21 counties used the registry with data on over 12,500 patients with MS. This is estimated to cover approximately 90% of all patients with MS in Finland [6]. The content of the registry is described in detail elsewhere [5]. All patients registered in the clinician-based registry can use the patient interface, called MyMS (patient interface of the Finnish MS registry), which can be accessed with the national Finnish electronic authorization. A total of 1201 patients with MS were reported to have recorded data into MyMS by the beginning of 2023 [6]. The content of the patient interface is described in our previous studies [6,7]. MyMS has existed since 2017 with the possibility to record background and lifestyle factors, suspected relapses, medications, need for assistance and social support, rehabilitation, and other diseases. Furthermore, 3 self-rating questionnaires, the Fatigue Severity Scale, the Multiple Sclerosis Impact Scale-29, and the 15D QoL scale have been available for users to assess their conditions since 2017 [6].

### Registry-Based Protocol for Screening of Fatigue and Cognitive Problems

The protocol for registry-based evaluation of cognitive problems and fatigue, along with the patient-perceived disability and QoL, was accepted by the Expert Academic Advisory Board of the Finnish MS registry. The protocol has been available since the beginning of 2021. The annual protocol includes the SDMT [15] as well as the following PROMs: the Patient-Reported Expanded Disability Status Scale (PREDSS) [16,17], the Visual Analog Scales (VASs) on neurological symptoms, the Euro QoL-5 dimensions [18], the Multiple Sclerosis Neuropsychological Questionnaire (MSNQ) [19,20], and the Fatigue Scale for Motor and Cognitive Functions (FSMC) [20,21]. The questionnaires are scored according to the standardized procedures of the instruments. The content of the protocol is described in Table 1.

**Table 1.** The annual protocol for screening and management of fatigue and cognitive problems by using the Finnish multiple sclerosis (MS) registry.

Assessment tool	Domain assessed	Assessment method / scoring	Role in the protocol
Symbol Digit Modalities Test (SDMT) in clinical MS registry	Cognitive processing speed	Assessed by a nurse; the patient matches symbols with digits in 90 seconds.	Screens for cognitive impairment. Neuropsychological assessment is recommended if the score declines by more than 10% or 4 points compared with baseline or previous assessment.
Patient-Reported Expanded Disability Status Scale (PREDSS) in MyMS^a^	Disease severity	Patient-reported evaluation of disease severity on a scale from 0 to 9, corresponding to the clinician-rated Expanded Disability Status Scale (EDSS).	Provides a perspective on patient-perceived disease severity.
Visual Analog Scales on Neurological Symptoms (VAS) in MyMS	Neurological symptoms	Patient-reported evaluation of symptom severity using a 0 to 100 scale, where 100 indicates the worst possible symptom.	Provides perspective on patient-perceived symptom severity. VAS mood score can be used to control the effect of mood on fatigue and cognition.
Euro Quality of Life-5 Dimension (EQ-5D) in MyMS	Quality of life	Patient-reported QoL evaluation using an index score from 0 to 1, where 1 indicates perfect QoL.	Provides perspective on patient-perceived quality of life.
Fatigue Scale for Motor and Cognitive Functions (FSMC) in MyMS	Motor, cognitive, and overall fatigue	Patient-reported; consists of 10 items on motor fatigue and 10 on cognitive fatigue, scored from 1 (totally disagree) to 5 (totally agree). Motor and cognitive subscores up to 50 each, total score up to 100.	Provides perspective on patient-perceived fatigue. Offers feedback to the patient including score interpretations and practical guidance for managing motor and cognitive fatigue in daily life.
Multiple Sclerosis Neuropsychological Questionnaire (MSNQ) in MyMS	Cognitive problems	Patient-reported; consists of 15 items assessing cognitive problems, each scored from 0 (does not occur) to 4 (very often/very disruptive), yielding a total score ranging from 0 to 60.	Provides perspective on patient-perceived cognitive problems. Offers feedback to the patient including score interpretation and practical guidance for managing cognitive difficulties in daily life.

a MyMS: patient interface of the Finnish MS registry.

According to the Invisible Symptoms protocol, patients with MS were familiarized with MyMS by the MS nurses in the hospital clinics. Educational material was offered for the nurses. Patients were provided with a leaflet containing instructions on how to use MyMS (Multimedia Appendix 1).

### Study Sample and Statistical Analysis

A preliminary sample collected from 5 well-being services counties between the beginning of 2021 and the end of 2022 was analysed. Date imputation, as the middle of the month or year, was used for clinical variables with partial dates. Date imputation was not needed for patient-reported data. The date of the first data entry in the patient interface was considered the index date to calculate age and disease duration. Descriptive statistics for demographics and PRO measures included means, SDs, medians, and IQRs for continuous variables, and counts and proportions for categorical variables. In addition, the proportion of missing data was reported.

For the correlation analysis, each pair of samples was created by matching the earliest dates of 2 PRO recordings that occurred within 28 days of one another. Correlation coefficients and testing were based on Pearson’s product-moment correlation. For controlling and checking the false discovery rate, the Benjamini-Hochberg procedure was used as a correction for multiple comparisons. *P* values under .05 after the correction were considered significant. All data analyses were done using RStudio (version 2021.09.1; Posit, PBC).

### Ethical Considerations

According to Finnish legislation, the approval of an ethical committee was not required as the study was a noninterventional registry study in which patients were not contacted. Patients permit the use of the data for research purposes including the secondary use when they log into the registry. Patients did not get any compensation for participation in the study. All used data were permitted via Finnish Social and Health Data Permit Authority Findata, which is responsible for data permits considering secondary use of data with multiple data sources and registry keepers (permission numbers THL/1809/14.02.00/2022 and THL/2104/14.05.00/2023 findata-rems-2023). Clinical data such as age at diagnosis, course of disease, disease duration, and Expanded Disability Status Scale (EDSS) [22] were extracted from the clinical MS registry and PRO data from the patient interface. All the data was analyzed in pseudonymized form, and in audited secure processing environment. Only anonymized information can be exported from the secure processing environment, and patient-level data is not publicly available to either authors or third-parties.StellarQ Ltd is the data processor for all data extracted.

## Results

The Invisible Symptoms protocol was available in 17 out of 21 Finnish well-being services counties at the time of the analysis. For the purposes of the study, we evaluated a sample from 5 Finnish well-being services counties: Southwest Finland, Satakunta, Pirkanmaa, Central Finland, North Savo. The population in these 5 counties represents approximately 30% of the total population in Finland and approximately 40% of the national MS population. The 5 counties represent areas with different population density and prevalence of MS. The implementation of the protocol was voluntary and depended on the resources the clinics had to administer the SDMT and introduce MyMS to the patients.

Altogether, 430 patients in the 5 well-being services counties had recorded data related to the Invisible Symptoms protocol into MyMS. The demographic and clinical variables of the protocol sample are listed in Table 2. The mean age of the patients at the first PRO recording was 42.5 years, the median disease duration was 4.1 years, and the median EDSS score was 2.5. Over 80% of patients were women (357 out of 430) and had a relapsing-remitting course of the disease (354 out of 430).

**Table 2.** Demographic and disease-related variables of the patients with data related to the Invisible Symptoms protocol.

Variable	Protocol sample (n=430)
Age at MS^a^ onset (years)	
Mean (SD)	33.1 (10.18)
Missing data, n (%)	108 (25.1)
Age at MS diagnosis (years), mean (SD)	35.5 (10.13)
Age at first PRO^b^ recording (years), mean (SD)	42.5 (10.28)
Sex (female), n (%)	357 (83.0)
Disease course, n (%)	
RRMS^c^	354 (82.3)
SPMS^d^	20 (4.7)
PPMS^e^	22 (5.1)
UNS^f^	34 (7.9)
Disease duration (years), median (Q1-Q3)	4.1 (0.6-11.3)
EDSS^g^	
Median (IQR)	2.5 (2.0-3.5)
Missing data, n (%)	50 (12)
Education (total years)	
Mean (SD)	14.3 (2.73)
Missing data, n (%)	118 (27.4)
Smoking (yes), n (%)	
Number	39 (10)
Missing data	24 (6)

a MS: multiple sclerosis.

b PRO: patient-reported outcome.

c RRMS: relapsing-remitting MS.

d SPMS: secondary progressive MS.

e PPMS: primary progressive MS.

f UNS: unspecified disease course.

g EDSS: Expanded Disability Status Scale.

Of the 430 patients, 349 (81.2%) patients have filled out the PREDSS with a mean score of 2.9 (SD 1.67) (Table 3). Table 3 summarizes the coverage of data on the SDMT as well as the PROMs related to the Invisible Symptoms protocol. The SDMT assessment was performed on 86 (20%) patients. The PROM on fatigue, the FSMC, was completed by 76.5% (329 out of 430) of the patients, whereas the PROM on cognitive problems, the MSNQ, was completed by 40.0% (172 out of 430). When a patient completes the FSMC, they receive a feedback report summarizing the results. The report also includes information about fatigue, along with practical tips for managing it in daily life. An example of the feedback report is presented as the Multimedia Appendix 2. A similar report is available after completing the MSNQ, summarizing the questionnaire results and providing tips for managing cognitive problems.

**Table 3.** Results on the Symbol Digit Modalities Test (SDMT) and the patient-reported measures of disability, quality of life, fatigue, cognition, and mood (n=430).

Variable	Total, n (%)	Mean (SD)	Median (IQR)
Patient Reported Expanded Disability Status Scale score (PREDSS)^a^	349 (81.2)	2.9 (1.67)	3.0 (2.0-4.0)
SDMT score	86 (20)	49.0 (13.56)	49.0 (40.8-56.8)
VAS^b^ scores, neurological symptoms			
Fatigue	319 (74.2)	45.2 (27.78)	42.0 (20.0-70.0)
Cognition	295 (68.6)	34.1 (25.96)	30.0 (10.5-52.0)
Mood	264 (61.4)	25.9 (26.10)	15.0 (6.8-37.2)
Euro Quality of Life-5D score^c^	297 (69.1)	0.8 (0.19)	0.8 (0.7-0.9)
FSMC^d^ scores			
Total	329 (76.5)	63.0 (22.49)	68.0 (47.0-80.0)
Motor fatigue	337 (78.4)	31.6 (11.43)	34.0 (24.0-41.0)
Cognitive fatigue	331 (77.0)	31.5 (11.68)	32.0 (23.0-41.0)
MSNQ^e^ scores			
Total score	172 (40.0)	35.3 (9.39)	35.0 (28.0-41.2)
Memory and learning	215 (50.0)	2.2 (0.74)	2.3 (1.7-2.7)
Attention	291 (67.7)	2.4 (0.85)	2.3 (1.8-3.0)
Verbal, problem-solving, and behavior	199 (46.3)	2.0 (0.64)	2.0 (1.5-2.3)

a PREDSS 0‐9 where 0 stands for no disability and 9 for bedridden most of the time.

b VAS: Visual Analog Scale. VAS scores where 0 stands for no problems and 100 for worst possible problems.

c EQ-5D: Euro Quality of Life–5 Dimension. Higher scores refer to better quality of life in EQ-5D (range 0‐1).

d FSMC: Fatigue Scale for Motor and Cognitive Functions. Higher scores refer to more severe symptoms/problems in the FSMC (total score range 20‐100, subscore range 10‐50).

e MSNQ: Multiple Sclerosis Neuropsychological Questionnaire. Higher scores refer to more severe/frequent cognitive problems in MSNQ (total score range 0‐60, subscores are means and range from 0 to 4).

**Figure 1.**
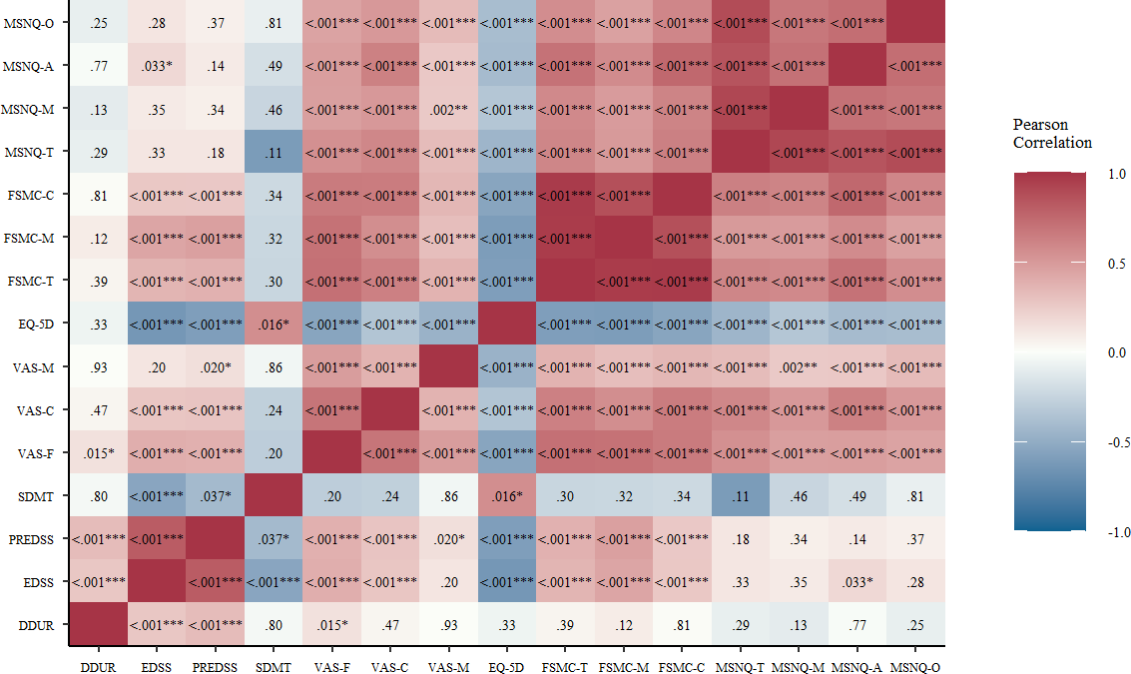
Correlation analysis of DDUR, disability (EDSS and PREDSS), SDMT performance, and PROMs on quality of life, fatigue, cognition, and mood. DDUR: disease duration; EDSS: Expanded Disability Status Scale; EQ-5D: Euro Quality of Life–5 Dimension; FSMC: Fatigue Scale for Motor and Cognitive Functions; MSNQ: Multiple Sclerosis Neuropsychological Questionnaire; PREDSS: Patient Reported Expanded Disability Status Scale; SDMT: Symbol Digit Modalities Test; VAS: Visual Analog Scale. *P<.05, **P<.01, ***P<.001.

In the present sample, a significant relationship was observed between disease duration and both the EDSS and the PREDSS (Figure 1). Disease duration did not show a significant relationship with any of the measures on mood, fatigue, cognition, or QoL except for the VAS-F. The EDSS correlated significantly with the SDMT, the VAS scores on fatigue and cognition, QoL, FSMC scores, and attentional items of the MSNQ. The PREDSS showed a correlation pattern similar to that of the EDSS. In contrast to the EDSS, it also correlated significantly with the VAS-M, but not with the MSNQ scores. The SDMT did not correlate significantly with the self-reports on mood, fatigue, or cognition. Instead, a significant correlation was observed between the SDMT and QoL. The VAS scores on fatigue, cognition, and mood correlated consistently with each other and the Euro Quality of Life–5 Dimension. Both the FSMC and MSNQ scores showed significant correlations with self-reports on fatigue, cognition, mood, and QoL.

## Discussion

The Finnish invisible symptom initiative presents a protocol for registry-based screening and management of fatigue and cognitive concerns. It includes an annual assessment of processing speed using the SDMT, as well as patient-reported measures of disease severity, QoL, and symptoms, with an emphasis on cognitive symptoms and fatigue. For symptom management purposes, patients are provided with a feedback report on self-perceived fatigue based on the FSMC and on self-perceived cognitive problems based on the MSNQ. Our sample from 5 Finnish well-being services counties shows that the implementation of the protocol has been gradual, and patient-reported assessments have been conducted more frequently than objective assessments of processing speed. The descriptive statistics of the present sample support earlier findings on the important role of fatigue and cognitive symptoms in QoL [7,10,12,13,23].

Our protocol for the screening and management of problems and fatigue aligns with the recommendations by Kalb et al [24] for baseline screening and annual follow-up of cognitive performance in MS using the SDMT. The SDMT has been introduced as the most sensitive measure of cognitive performance and processing speed in MS [25]. The Finnish validation of the brief international cognitive assessment in MS showed that the SDMT was the most sensitive measure, also in Finnish patients with MS [20]. In the same study, the MSNQ and the FSMC were found to be suitable for self-evaluation of fatigue and cognitive symptoms, provided that the effects of mood state are adequately controlled. To assess patient perspective, the MSNQ and the FSMC were thus implemented into the patient interface of the Finnish MS registry. The VAS score on mood was used to control the effects of mood on fatigue and cognitive concerns.

Only 20% (86 out of 430) of the patients in the present sample were assessed with the SDMT. The mean SDMT score in the present registry-based sample was higher than that observed in the Finnish validation study with older and more disabled patients [20] but in line with international studies with patients of similar age and EDSS [26-29]. Over 50% (291 out of 430) of the patients of the present sample had completed the MSNQ, and over 70% (337 out of 430) of the patients had completed the FSMC, at least partially. Based on the FSMC [21], the patients of the present sample reported severe overall fatigue and moderate motor and cognitive fatigue. Based on the MSNQ [19], they also perceived at least occasional problems with cognitive functions. The patients of the present sample reported more fatigue and cognitive concerns than the patients in the Finnish validation study [20]. Further, the FSMC scores of the present sample were higher than those in another Finnish study involving patients with relapsing-remitting MS [10]. The present sample is likely overrepresented by patients with pronounced fatigue and cognitive symptoms, as these individuals were probably more often instructed to complete the questionnaires to support symptom management.

In the present sample, physical disability as measured by either the EDSS or the PREDSS correlated with all the self-report measures used, except the MSNQ. On the contrary, disease duration did not have a significant relationship with the self-reports, certifying the earlier findings that invisible symptoms may emerge at any phase of the disease [30]. The SDMT correlated with both the EDSS and the PREDSS. The SDMT did not show a significant relationship with the self-report of depression. On the contrary, self-reports on fatigue and cognitive problems correlated significantly with those on depression. These results are in line with earlier findings that concerns with fatigue, cognition, and mood are interrelated and should all be considered in the screening and search for optimal treatment methods [11,14,19,20].

In the Invisible Symptoms initiative, feedback reports based on the patient’s answers in the MSNQ and the FSMC were developed. Digital self-management tools could be further developed into web-based rehabilitation programs that integrate assessments, feedback, and strategies with individual goal setting and professional counseling. Positive effects on the management of cognitive symptoms in MS have been demonstrated not only through neuropsychological and cognitive rehabilitation, but also through the use of digital applications [31-33]. Especially cost-effective, individualized, and holistic intervention with an input from HCP has been called for [11].

This study has several limitations. The procedure for screening and managing fatigue and cognitive concerns has been in use for only 2 years and is not yet fully implemented across all regions, resulting in a relatively small sample limited to 5 of Finland’s 21 wellbeing services counties—though these account for approximately 40% of the national MS population. Adoption of the protocol has likely been slowed by limited resources for conducting the SDMT and challenges in interpreting and integrating PROM data into routine clinical decision-making. Additionally, the patient interface has so far been available only as a web-based tool, which may have reduced usability; a mobile app is currently under development in response to patient feedback, particularly from younger individuals. The sample may also over-represent individuals with fatigue and cognitive symptoms, as participation is voluntary and allows patients to choose which questionnaires to complete. It is also unknown whether patients have engaged with the feedback provided. Despite these limitations, the model offers clinics a structured means to guide annual assessment and symptom management through the digital registry. Ideally, this approach would be applied systematically and include both PROMs and the SDMT, which is internationally recommended for evaluating cognitive function in people with MS.

Comprehensive management of invisible symptoms requires effective communication between HCPs and patients [34,35]. The Finnish Invisible Symptoms initiative provides a systematic approach to addressing patient-perceived cognitive problems and fatigue. Cognitive performance and patient-perceived symptoms, including fatigue and cognitive concerns, can be assessed annually using validated tools, with results accessible to patients via the patient interface and to HCPs via the clinician interface. According to a European survey published in 2019 [1], only 7 out of 19 identified MS registries include patient-derived measures. Digital registries can serve as platforms for patient-reported data, empowering individuals to take an active role in managing their disease, adhering to treatment, and making beneficial lifestyle choices. As stated by Lakin et al [34], by pairing clinical knowledge with an understanding and consideration of the patient perspective, HCPs are equipped to foster patient-centered dialogue that encourages shared decision-making and high-quality care. The need for innovative interventions addressing invisible symptoms is increasingly recognized. Positive experiences with digital solutions have already been reported, highlighting the importance of continued development and evaluation [31,33,36].

## Supplementary material

10.2196/67990 Multimedia Appendix 1 Instruction leaflet describing the use of MyMS.

10.2196/67990 Multimedia Appendix 2 An example of the feedback report the patient receives after filling out the Fatigue Scale for Motor and Cognitive Functions (FSMC).

10.2196/67990 Checklist 1 STROBE checklist.

## Abbreviations

EDSS: Expanded Disability Status Scale
FSMC: Fatigue Scale for Motor and Cognitive Functions
FSS: Fatigue Severity Scale
HCP: health care professional
MS: multiple sclerosis
MSNQ: Multiple Sclerosis Neuropsychological Questionnaire
MyMS: patient-interface of the Finnish multiple sclerosis registry
PREDSS: Patient Reported Expanded Disability Status Scale
PRO: patient-reported outcome
PROM: patient-reported outcome measure
QoL: quality of life
SDMT: Symbol Digit Modalities Test
VAS: Visual Analog Scale
