# Supplementary figures and images for: Finnish Registry-Based Protocol for Screening and Management of Fatigue and Cognitive Problems in Multiple Sclerosis: Observational Study
Source: JMIR Hum Factors. 2025 Aug 22;12:e67990. doi: 10.2196/67990 (PMC12373260; doi:10.2196/67990)

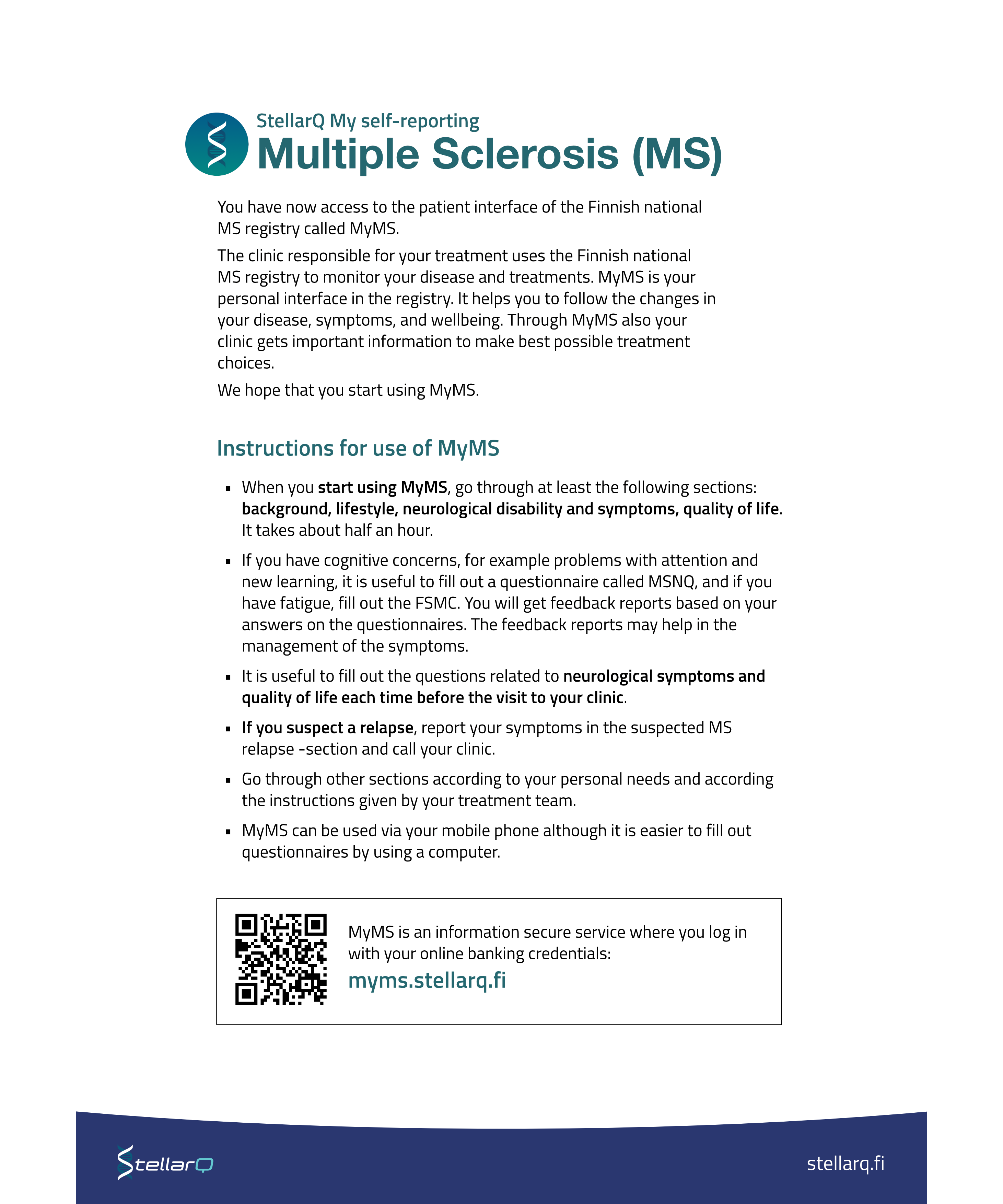

Supplement: Multimedia Appendix 1 [file humanfactors-v12-e67990-s001.png]

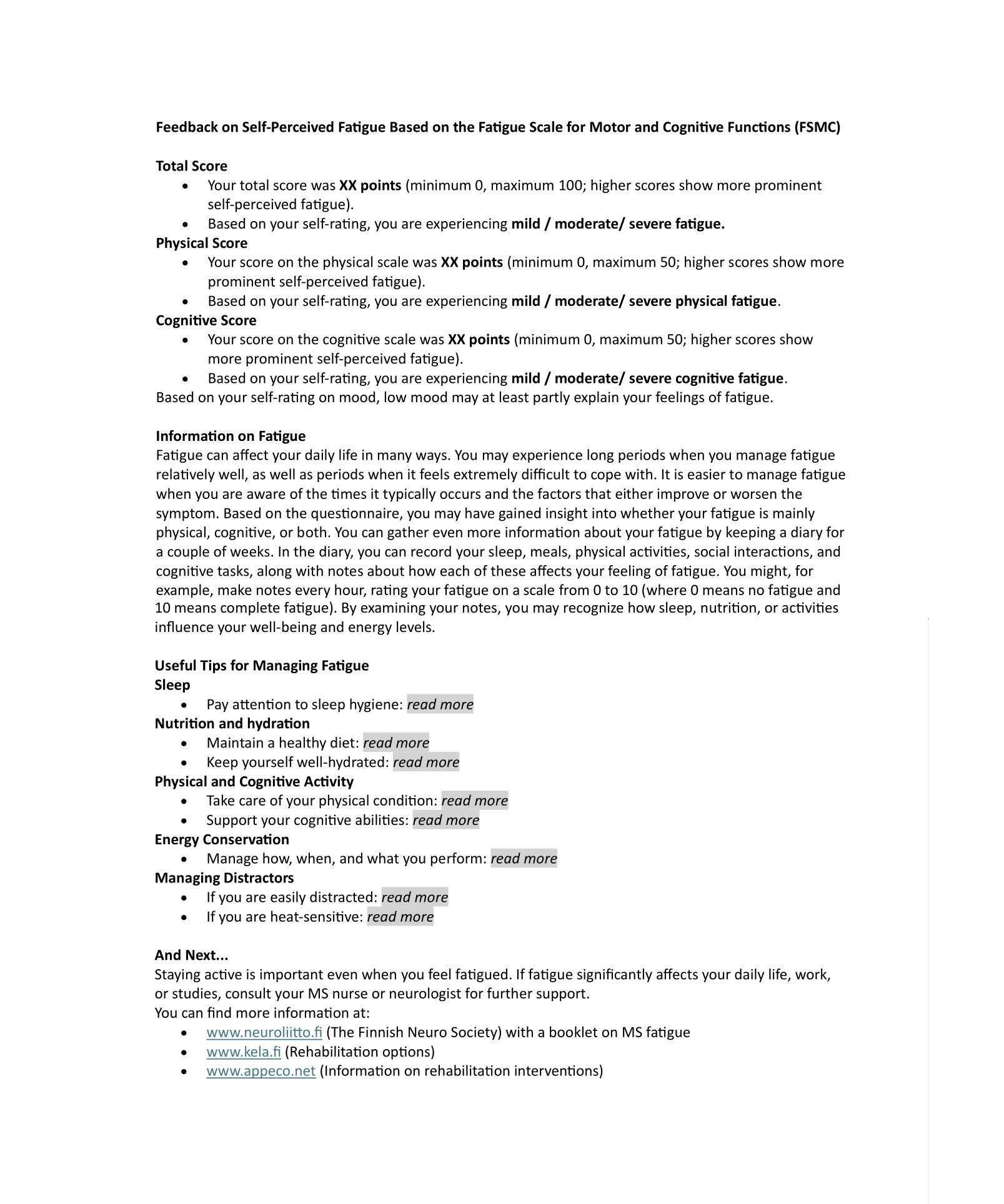

Supplement: Multimedia Appendix 2 [file humanfactors-v12-e67990-s002.png]
